# Supplementary material for: Dietary Fibre Impacts the Texture of Cooked Whole Grain Rice
Source: Foods. 2023 Feb 20;12(4):899. doi: 10.3390/foods12040899 (PMC9957187; doi:10.3390/foods12040899)
Supplement: Supplementary file 1 [file foods-12-00899-s001.zip › Supplementary Figures.pdf]

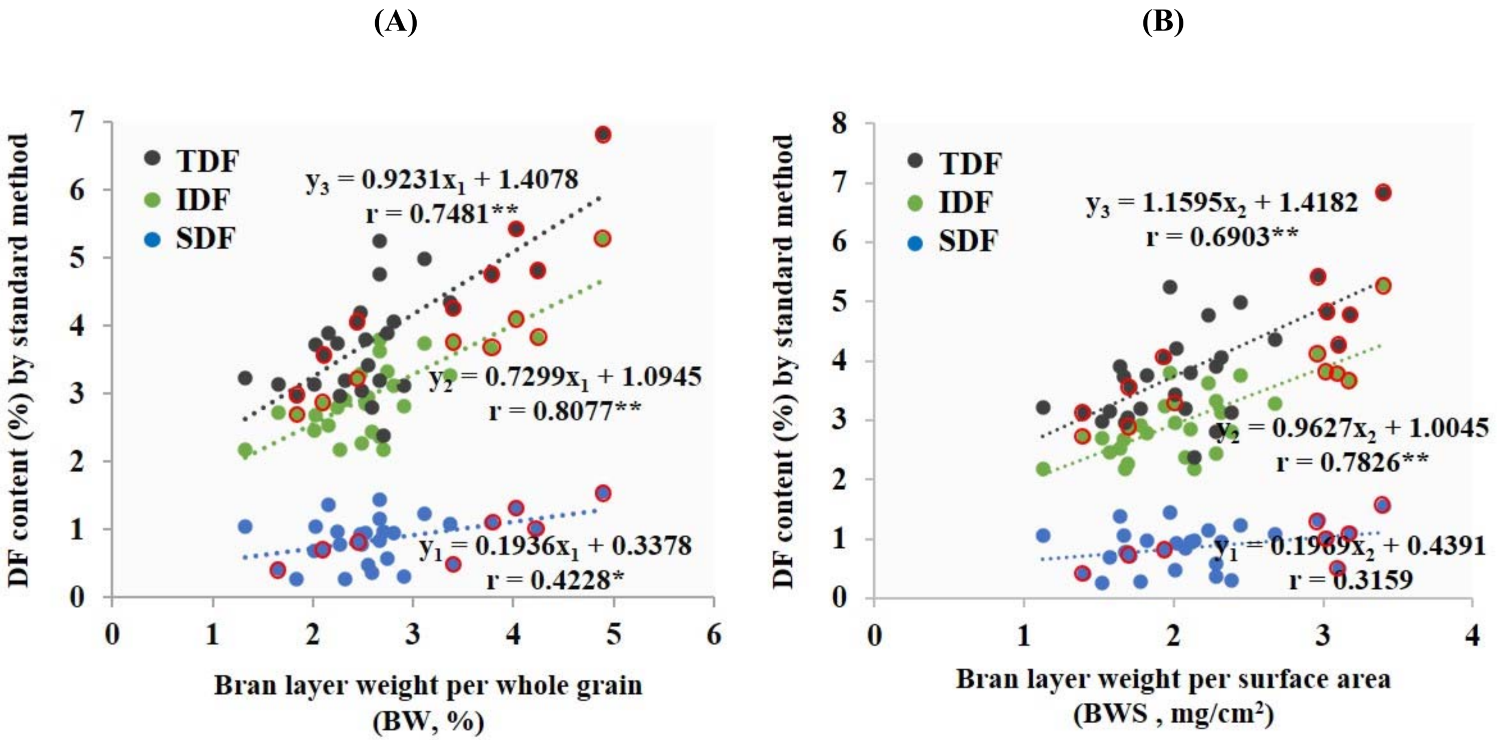

Figure S1. Linear regression between the percentage of dietary fibre content: SDF, IDF and, TDF, and **(A)** the percentage of bran layer weight without germ in whole grain rice (BW, g/100 g) determined by the alkaline method or **(B)** the bran layer weight without germ per surface area (BWS, mg/cm^2). Rice varieties with ASV equal to exactly 1 are shown as circles with red border. * Correlation is significant at the 0.05 level; ** Correlation is significant at the 0.01 level.

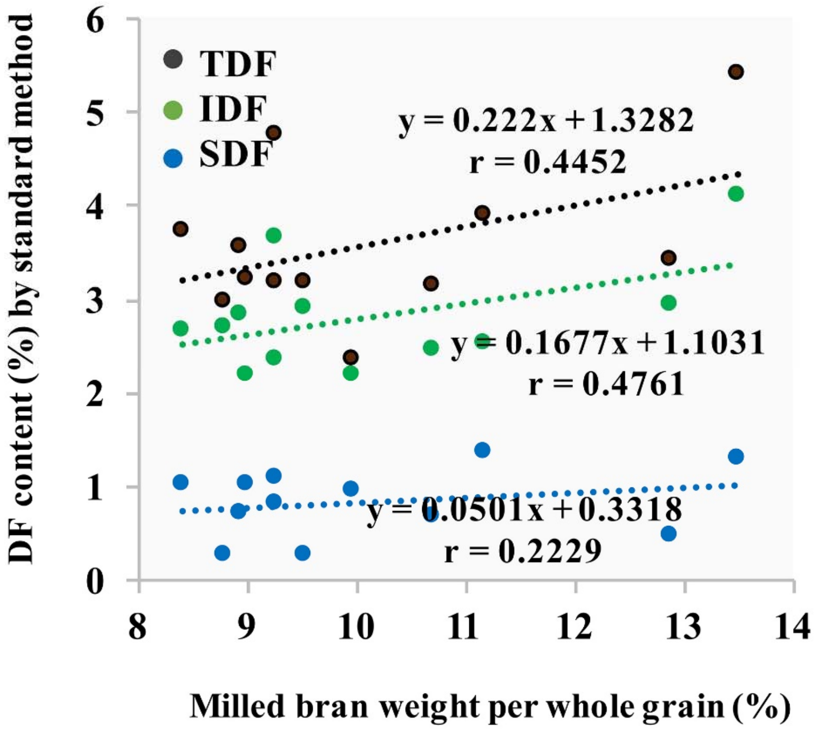

Varieties	Bran (%)
PK4#20A09	8.81
PK4#78A03	8.94
RD43	9.55
RD15	12.91
PK1	8.42
M7881	9.97
SLK	9.00
JHN	10.73
RB	11.19
NHN	9.29
KH	13.51
RD6	9.27

Figure S2. Linear regression between the percentage of dietary fibre content: SDF, IDF and, TDF, and the percentage of the rice bran weight determined by the milling method.
