# Supplementary material for: Dietary Fibre Impacts the Texture of Cooked Whole Grain Rice
Source: Foods. 2023 Feb 20;12(4):899. doi: 10.3390/foods12040899 (PMC9957187; doi:10.3390/foods12040899)
Supplement: Supplementary file 1 [file foods-12-00899-s001.zip › Supplementary Tables.pdf]

Table S1. Rice bran traits of whole grain rice samples.

Varieties	Bran layer without germ			Bran layer with germ		
	BW (g/100 g)	BWG (mg/g)	BWS (mg/cm²)	BW (g/100 g)	BWG (mg/g)	BWS (mg/cm²)
66B09	$2.75 \pm 0.12^{g-j}$	$27.45 \pm 1.23^{g-j}$	$2.28 \pm 0.10^{h-j}$	$5.97 \pm 0.25^{j-l}$	$59.67 \pm 2.47^{j-l}$	$4.96 \pm 0.21^{j-l}$
PK4#20A09	$1.84 \pm 0.06^{a-c}$	$18.44 \pm 0.65^{a-c}$	$1.52 \pm 0.05^{a-c}$	$4.96 \pm 0.12^{d-g}$	$49.59 \pm 1.15^{d-g}$	$4.09 \pm 0.09^{e-h}$
16F35	$2.58 \pm 0.08^{e-j}$	$25.85 \pm 0.79^{e-j}$	$2.28 \pm 0.07^{h-j}$	$5.20 \pm 0.23^{f-h}$	$51.95 \pm 2.34^{f-h}$	$4.59 \pm 0.21^{h-k}$
MU2-00005	$2.43 \pm 0.04^{d-i}$	$24.34 \pm 0.36^{d-i}$	$1.94 \pm 0.03^{c-h}$	$4.36 \pm 0.22^{b-d}$	$43.57 \pm 2.20^{b-d}$	$3.48 \pm 0.18^{a-d}$
PK4#117A08	$2.91 \pm 0.12^{i-k}$	$29.06 \pm 1.20^{i-k}$	$2.38 \pm 0.10^{i,j}$	$5.01 \pm 0.05^{e-g}$	$50.13 \pm 0.62^{e-g}$	$4.11 \pm 0.05^{e-h}$
PK4#78A03	$2.10 \pm 0.10^{c-f}$	$21.03 \pm 1.02^{c-f}$	$1.71 \pm 0.08^{b-f}$	$5.46 \pm 0.10^{g-j}$	$54.60 \pm 1.05^{g-j}$	$4.44 \pm 0.09^{g-j}$
DGR	$2.27 \pm 0.10^{c-f}$	$22.69 \pm 1.01^{c-f}$	$1.68 \pm 0.07^{b-d}$	$4.44 \pm 0.83^{b-e}$	$44.44 \pm 8.39^{b-e}$	$3.28 \pm 0.62^{a-c}$
M9997	$2.24 \pm 0.13^{c-g}$	$22.38 \pm 1.25^{c-g}$	$1.82 \pm 0.10^{b-g}$	$4.74 \pm 0.29^{c-f}$	$47.41 \pm 2.86^{c-f}$	$3.86 \pm 0.23^{d-f}$
BMT	$2.48 \pm 0.07^{d-i}$	$24.83 \pm 0.74^{d-i}$	$1.69 \pm 0.05^{b-f}$	$5.29 \pm 0.08^{f-i}$	$52.87 \pm 0.75^{f-i}$	$3.61 \pm 0.05^{b-e}$
KTH	3.39 ± 0.31^{o}	33.89 ± 3.10^{o}	$3.11 \pm 0.28^{l,m}$	5.65 ± 0.35^{a}	56.52 ± 3.48^{a}	$5.18 \pm 0.32^{l,m}$
RD43	$2.32 \pm 0.04^{c-h}$	$23.16 \pm 0.35^{c-h}$	$1.78 \pm 0.03^{b-f}$	$5.79 \pm 0.08^{i-k}$	$57.85 \pm 0.80^{i-k}$	$4.45 \pm 0.06^{g-i}$
RD15	$2.55 \pm 0.06^{d-j}$	$25.45 \pm 0.55^{d-j}$	$2.01 \pm 0.04^{d-i}$	$5.75 \pm 0.21^{h-k}$	$57.50 \pm 2.08^{h-k}$	$4.55 \pm 0.16^{g-k}$
PNL80	$2.81 \pm 0.15^{h-j}$	$28.12 \pm 1.46^{h-j}$	$2.31 \pm 0.12^{h-j}$	$5.51 \pm 0.23^{g-k}$	$55.12 \pm 2.34^{g-k}$	$4.54 \pm 0.19^{g-k}$
PK1	$2.02 \pm 0.14^{b-d}$	$20.23 \pm 1.37^{b-d}$	$1.67 \pm 0.11^{b-e}$	$4.99 \pm 0.14^{e-g}$	$49.85 \pm 1.37^{e-g}$	$4.12 \pm 0.11^{e-h}$
KDML105	$2.53 \pm 0.49^{d-i}$	$25.29 \pm 4.88^{d-i}$	$2.11 \pm 0.41^{f-i}$	$5.33 \pm 0.39^{g-i}$	$53.27 \pm 3.90^{g-i}$	$4.45 \pm 0.33^{g-j}$
HS	$3.37 \pm 0.04^{k,l}$	$33.67 \pm 0.43^{k,l}$	$2.68 \pm 0.03^{j,k}$	$5.04 \pm 0.15^{e-g}$	$50.38 \pm 1.49^{e-g}$	$4.01 \pm 0.12^{d-g}$
PTN1	$2.48 \pm 0.12^{d-i}$	$24.80 \pm 1.22^{d-i}$	$2.02 \pm 0.10^{d-i}$	$5.22 \pm 0.06^{f-i}$	$52.21 \pm 0.61^{f-i}$	$4.26 \pm 0.05^{f-h}$
M7881	$2.71 \pm 0.35^{f-j}$	$27.13 \pm 3.50^{f-j}$	$2.14 \pm 0.28^{e-i}$	6.41 ± 0.25^{l}	64.13 ± 2.46^{l}	$5.06 \pm 0.19^{k,l}$
MU2-42	$2.67 \pm 0.19^{e-j}$	$26.66 \pm 1.95^{e-j}$	$1.98 \pm 0.14^{c-i}$	$6.14 \pm 0.10^{k,l}$	$61.42 \pm 1.02^{k,l}$	$4.55 \pm 0.08^{h-j}$
SL	1.33 ± 0.04^{a}	13.30 ± 0.40^{a}	1.12 ± 0.03^{a}	3.74 ± 0.04^{a}	37.39 ± 0.32^{a}	$3.16 \pm 0.03^{a,b}$
909-10-3	$2.67 \pm 0.14^{f-j}$	$26.67 \pm 1.39^{f-j}$	$2.24 \pm 0.12^{g-i}$	$4.24 \pm 0.22^{a-c}$	$42.36 \pm 2.25^{a-c}$	$3.55 \pm 0.19^{a-e}$
JHN	$2.02 \pm 0.09^{b-e}$	$20.17 \pm 0.93^{b-e}$	$1.83 \pm 0.08^{b-h}$	$4.52 \pm 0.04^{b-e}$	$45.24 \pm 0.42^{b-e}$	$3.52 \pm 0.03^{a-d}$
HCS	$1.66 \pm 0.01^{a,b}$	$16.59 \pm 0.09^{a,b}$	$1.40 \pm 0.01^{a,b}$	$4.01 \pm 0.12^{a,b}$	$40.05 \pm 1.02^{a,b}$	$3.38 \pm 0.09^{a-c}$
HLN	$3.11 \pm 0.09^{j,k}$	$31.12 \pm 0.87^{j,k}$	$2.45 \pm 0.07^{i,j}$	$6.10 \pm 0.11^{k,l}$	$60.97 \pm 1.14^{k,l}$	$4.80 \pm 0.09^{j-l}$
RB	$2.15 \pm 0.05^{b-f}$	$21.55 \pm 0.49^{b-f}$	$1.64 \pm 0.04^{b-d}$	$4.02 \pm 0.07^{a,b}$	$40.23 \pm 0.71^{a,b}$	3.06 ± 0.05^{a}
SYN	4.24 ± 0.16^{m}	42.35 ± 1.57^{m}	$3.03 \pm 0.11^{k-m}$	$5.25 \pm 0.03^{f-i}$	$52.48 \pm 0.29^{f-i}$	$3.76 \pm 0.02^{c-f}$
NHN	$3.78 \pm 0.41^{l-m}$	$37.83 \pm 4.10^{l-m}$	$3.19 \pm 0.35^{l,m}$	$6.11 \pm 0.43^{k,l}$	$61.10 \pm 4.29^{k,l}$	$5.14 \pm 0.36^{l,m}$
KH	4.03 ± 0.12^{m}	40.32 ± 1.18^{m}	$2.97 \pm 0.09^{k,l}$	$7.69 \pm 0.29^{f-i}$	$76.93 \pm 2.87^{f-i}$	5.66 ± 0.21^{l}
MU1-2313	4.89 ± 0.29^{n}	48.86 ± 2.88^{n}	3.41 ± 0.20^{m}	10.43 ± 0.05^{m}	104.30 ± 0.53^{m}	7.28 ± 0.04^{m}
RD6	$2.67 \pm 0.33^{f-j}$	$26.72 \pm 3.33^{f-j}$	$2.08 \pm 0.26^{e-i}$	$6.16 \pm 0.49^{k,l}$	$61.61 \pm 4.88^{k,l}$	$4.79 \pm 0.38^{i-l}$

Results are the means ± SE on dry basis. Values with different letters in the same column are significantly different with $P < 0.05$. The weight of bran layer without germ or with germ, determined by alkaline method are presented as bran layer weight/whole grain rice: BW, g/100 g and BWG, mg/g, and presented as bran layer weight/unit of surface area: BWS, mg/cm².

Table S2. Physical characteristics of whole grain rice samples.

Varieties	^{a}Alkaline test	^{b}Shape				^{c}FKW (g)	^{d}Surface area	
		L (cm)	W (cm)	T (cm)	L : W		s (cm^2/kernel)	S (cm^2/g)
66B09	2.40	0.75 ± 0.01^{j-l}	0.22 ± 0.00^{o-q}	0.17 ± 0.00^{h-j}	3.45 ± 0.04^{d,e}	1.067 ± 0.004^{k-m}	0.257 ± 0.007^{j}	12.03 ± 0.31$^{b-d,f}$
PK4#20A09	4.50	0.71 ± 0.02^{e-h}	0.23 ± 0.01^{r}	0.18 ± 0.00^{k,l}	3.07 ± 0.12^{a}	1.090 ± 0.006^{l,m}	0.265 ± 0.007^{j}	12.13 ± 0.31^{b-f}
16F35	1.70	0.75 ± 0.01^{j-l}	0.22 ± 0.00^{p,q}	0.17 ± 0.00^{g-i}	3.46 ± 0.07^{d,e}	1.127 ± 0.005^{m}	0.255 ± 0.006^{j}	11.32 ± 0.25^{a,b}
MU2-00005	1.00	0.74 ± 0.01^{h-k}	0.19 ± 0.00^{c-e}	0.16 ± 0.00^{c,d}	3.87 ± 0.07^{j-m}	0.901 ± 0.012^{e-h}	0.226 ± 0.002^{f,g}	12.52 ± 0.10^{d-j}
PK4#117A08	3.40	0.76 ± 0.01^{l,m}	0.21 ± 0.00^{k-p}	0.17 ± 0.00^{j,k}	3.65 ± 0.04^{f-i}	1.068 ± 0.006^{k-m}	0.260 ± 0.002^{j}	12.19 ± 0.11^{c-g}
PK4#78A03	1.00	0.74 ± 0.02^{h-l}	0.20 ± 0.00^{d-l}	0.16 ± 0.00^{e-i}	3.71 ± 0.08^{g-k}	0.978 ± 0.004^{d-f}	0.241 ± 0.005^{g-i}	12.30 ± 0.28^{c-i}
DGR	1.25	0.70 ± 0.00^{d-g}	0.20 ± 0.01^{f-m}	0.17 ± 0.00^{i-k}	3.50 ± 0.11^{d-g}	0.877 ± 0.005^{c-h}	0.238 ± 0.005^{f-h}	13.54 ± 0.29^{j-l}
M9997	1.40	0.73 ± 0.01^{g-j}	0.21 ± 0.00^{l-p}	0.17 ± 0.00^{g-i}	3.48 ± 0.08^{d-f}	0.992 ± 0.003^{h-k}	0.244 ± 0.006^{h,i}	12.30 ± 0.28^{c-h}
BMT	1.63	0.75 ± 0.02^{i-l}	0.18 ± 0.00^{b}	0.15 ± 0.00^{b-d}	4.20 ± 0.12^{o}	0.750 ± 0.005^{b,c}	0.219 ± 0.008^{c-f}	14.66 ± 0.51^{n}
KTH	1.00	0.68 ± 0.01^{b-d}	0.21 ± 0.00^{m-p}	0.16 ± 0.00^{f-i}	3.23 ± 0.07^{a-c}	1.057 ± 0.007^{j-m}	0.230 ± 0.007^{f,g}	10.90 ± 0.33^{a}
RD43	2.75	0.75 ± 0.01^{k-m}	0.20 ± 0.00^{h-o}	0.17 ± 0.00^{j,k}	3.77 ± 0.05^{g-k}	0.994 ± 0.002^{h-l}	0.259 ± 0.006^{i,j}	13.01 ± 0.30^{g-k}
RD15	2.75	0.75 ± 0.02^{i-l}	0.20 ± 0.01^{c-j}	0.16 ± 0.01^{c-f}	3.78 ± 0.08^{h-l}	0.925 ± 0.003^{e-i}	0.234 ± 0.004^{f-h}	12.64 ± 0.20^{d-k}
PNL80	4.10	0.71 ± 0.01^{e-g}	0.20 ± 0.00^{g-k}	0.16 ± 0.00^{c-e}	3.53 ± 0.05^{d-g}	0.929 ± 0.005^{f-i}	0.226 ± 0.004^{f,g}	12.15 ± 0.24^{c-h}
PK1	3.75	0.76 ± 0.04^{k-m}	0.21 ± 0.01^{j-p}	0.17 ± 0.01^{h-k}	3.65 ± 0.11^{e-j}	1.070 ± 0.005^{k-m}	0.259 ± 0.010^{j}	12.11 ± 0.45^{b-h}
KDML105	2.63	0.75 ± 0.03^{i-l}	0.20 ± 0.01^{g-n}	0.16 ± 0.01^{e-i}	3.66 ± 0.09^{e-j}	1.022 ± 0.008^{i-m}	0.244 ± 0.005^{h-j}	11.96 ± 0.24^{b-f}
HS	1.20	0.72 ± 0.01^{f-i}	0.20 ± 0.00^{c-h}	0.15 ± 0.00^{b,c}	3.68 ± 0.05^{g-i}	0.879 ± 0.006^{d-g}	0.221 ± 0.004^{d-f}	12.57 ± 0.23$^{e,g-j}$
PTN1	2.30	0.71 ± 0.01^{e-h}	0.20 ± 0.00^{e-i}	0.15 ± 0.00^{c}	3.58 ± 0.05^{e-i}	0.908 ± 0.004^{e-i}	0.223 ± 0.003^{d-f}	12.26 ± 0.19^{c-i}
M7881	2.00	0.79 ± 0.03^{m}	0.19 ± 0.01^{c-g}	0.16 ± 0.01^{c-g}	4.10 ± 0.10^{n,o}	0.956 ± 0.005^{f-k}	0.242 ± 0.009^{h-j}	12.67 ± 0.49$^{e, g-k}$
MU2-42	1.50	0.67 ± 0.01^{b,c}	0.19 ± 0.00^{c}	0.15 ± 0.00^{b}	3.56 ± 0.05^{e-h}	0.742 ± 0.004^{b}	0.200 ± 0.004^{b}	13.49 ± 0.27^{k,l}
SL	1.75	0.69 ± 0.01^{b-f}	0.20 ± 0.01^{f-m}	0.17 ± 0.01^{h-j}	3.44 ± 0.09^{c-f}	0.978 ± 0.005^{h-k}	0.231 ± 0.003^{e-h}	11.83 ± 0.15^{b,c}
909-10-3	1.60	0.67 ± 0.00^{b}	0.20 ± 0.00^{d-i}	0.15 ± 0.00^{b,c}	3.37 ± 0.05^{b-d}	0.872 ± 0.003^{d-g}	0.208 ± 0.004^{b,c}	11.92 ± 0.20^{b,c}
JHN	2.00	0.67 ± 0.01^{b-e}	0.19 ± 0.00^{b-d}	0.16 ± 0.00^{c-f}	3.61 ± 0.11^{e-i}	0.818 ± 0.001^{b-g}	0.210 ± 0.005^{b-d}	12.85 ± 0.28^{e-k}
HCS	1.00	0.73 ± 0.04^{g-l}	0.19 ± 0.01^{c-h}	0.16 ± 0.00^{d-h}	3.80 ± 0.16^{i-m}	0.980 ± 0.006^{h-j}	0.233 ± 0.007^{f-h}	11.86 ± 0.34^{b-d}
HLN	1.60	0.67 ± 0.01^{b}	0.19 ± 0.00^{c-f}	0.15 ± 0.00^{b,c}	3.50 ± 0.06^{d-g}	0.805 ± 0.001^{b-d}	0.205 ± 0.004^{b}	12.71 ± 0.25$^{e,g-j}$
RB	2.00	0.68 ± 0.01^{b-d}	0.18 ± 0.00^{b-d}	0.15 ± 0.00^{b,c}	3.89 ± 0.14^{d-i}	0.771 ± 0.001^{b-e}	0.203 ± 0.005^{b}	13.14 ± 0.29^{i-l}
SYN	1.00	0.62 ± 0.01^{a}	0.16 ± 0.00^{a}	0.14 ± 0.00^{a}	3.90 ± 0.08^{k-n}	0.582 ± 0.002^{a}	0.163 ± 0.002^{a}	13.97 ± 0.13^{l,m}
NHN	1.00	0.90 ± 0.01^{o}	0.22 ± 0.01^{n-q}	0.19 ± 0.01^{l}	4.12 ± 0.02^{m-o}	1.313 ± 0.007^{n}	0.312 ± 0.010^{l}	11.88 ± 0.36^{b-f}
KH	1.00	0.84 ± 0.01^{n}	0.21 ± 0.00^{i-p}	0.18 ± 0.01^{j,k}	4.02 ± 0.06^{l-o}	1.046 ± 0.011^{j-m}	0.284 ± 0.010^{k}	13.58 ± 0.49^{k,l}
MU1-2313	1.00	0.70 ± 0.01^{c-f}	0.20 ± 0.00^{c-h}	0.15 ± 0.01^{b}	3.55 ± 0.03^{d-g}	0.735 ± 0.006^{b}	0.211 ± 0.004^{b-e}	14.33 ± 0.30^{m,n}
RD6	4.00	0.71 ± 0.01^{e-h}	0.23 ± 0.00^{q,r}	0.16 ± 0.00^{c-g}	3.15 ± 0.11^{a,b}	0.946 ± 0.008^{g-i}	0.243 ± 0.003^{h-j}	12.85 ± 0.15^{h-k}

Results are the means ± SE on dry basis. Values with different letters in the same column are significantly different with $P < 0.05$. ^{a}Alkaline spreading test, the spreading value was rated visually on a 7-point numerical scale: 1 = Intact, 7= Greatly dispersed. ^{b} Shape: L, length; W, width; T, thickness; L : W, length : width ratio. ^{c}FKW, 50 kernel weight. ^{d}Surface area; s, surface area/kernel where S, surface area/g whole grain rice.

Table S3. Chemical compositions of whole grain rice samples.

Varieties	Colour	Amylose	Starch	[a]Dietary fibre			
				SDF	IDF	TDF	SDF:IDF
66B09	White	27.53 ± 0.05[a]	81.92 ± 0.52[g,h]	0.57 ± 0.03[a-e]	3.33 ± 0.02[e-g]	3.90 ± 0.01[e-h]	0.17 ± 0.01[a-e]
PK4#20A09	White	26.86 ± 0.04[b]	84.29 ± 0.46[i,j]	0.27 ± 0.02[a]	2.71 ± 0.38[a-e]	2.97 ± 0.40[a,b]	0.10 ± 0.01[a]
16F35	White	26.77 ± 0.11[b]	78.76 ± 0.15[c-e]	0.37 ± 0.05[a]	2.44 ± 0.33[a-c]	2.80 ± 0.29[a]	0.15 ± 0.04[a-d]
MU2-00005	White	26.73 ± 0.63[b]	83.12 ± 0.41[h-j]	0.82 ± 0.21[e-h]	3.23 ± 0.12[e-g]	4.05 ± 0.09[f-i]	0.25 ± 0.07[b-g]
PK4#117A08	White	26.69 ± 0.48[b]	80.92 ± 0.35[f,g]	0.31 ± 0.01[a]	2.82 ± 0.24[a-e]	3.13 ± 0.23[a-d]	0.11 ± 0.01[a]
PK4#78A03	White	25.74 ± 0.47[c]	84.56 ± 0.77[j]	0.71 ± 0.00[b-f]	2.86 ± 0.18[b-e]	3.57 ± 0.18[b-g]	0.25 ± 0.02[b-f]
DGR	White	25.01 ± 0.21[d]	83.51 ± 0.64[h-j]	0.78 ± 0.14[c-g]	2.18 ± 0.59[a]	2.96 ± 0.73[a,b]	0.36 ± 0.04[f-j]
M9997	White	23.91 ± 0.04[e]	78.12 ± 0.43[b-e]	0.96 ± 0.01[f-j]	2.79 ± 0.16[a-e]	3.75 ± 0.16[c-h]	0.34 ± 0.02[f-i]
BMT	White	22.48 ± 0.36[f]	79.64 ± 0.46[e,f]	0.79 ± 0.11[d-g]	2.26 ± 0.18[a,b]	3.05 ± 0.07[a-c]	0.35 ± 0.07[f-i]
KTH	White	21.73 ± 0.04[g]	79.05 ± 0.12[d-f]	0.49 ± 0.04[a-d]	3.77 ± 0.07[f-h]	4.26 ± 0.03[g-k]	0.13 ± 0.01[a,b]
RD43	White	19.98 ± 0.28[h]	75.83 ± 0.83[a]	0.27 ± 0.01[a]	2.92 ± 0.02[c-e]	3.19 ± 0.03[a-e]	0.09 ± 0.00[a]
RD15	White	18.23 ± 0.06[i]	84.26 ± 0.20[i,j]	0.48 ± 0.01[a-c]	2.95 ± 0.21[c-e]	3.43 ± 0.21[a-f]	0.16 ± 0.01[a-e]
PNL80	White	17.84 ± 0.00[i,j]	76.42 ± 0.79[a,b]	0.94 ± 0.06[f-j]	3.13 ± 0.47[d-f]	4.07 ± 0.53[f-j]	0.31 ± 0.03[f-h]
PK1	White	17.66 ± 0.21[i,j]	82.85 ± 0.16[h-j]	1.05 ± 0.01[g-k]	2.68 ± 0.23[a-e]	3.73 ± 0.24[c-h]	0.39 ± 0.03[h-j]
KDML105	White	17.57 ± 0.13[j,k]	83.28 ± 0.68[h-j]	0.95 ± 0.01[f-j]	2.85 ± 0.35[b-e]	3.80 ± 0.35[d-h]	0.34 ± 0.04[f-i]
HS	White	17.37 ± 0.19[j,k]	82.47 ± 1.06[g-i]	1.08 ± 0.01[g-l]	3.28 ± 0.13[e-g]	4.36 ± 0.12[h-l]	0.33 ± 0.02[f-i]
HD	White	16.98 ± 0.22[k,l]	80.90 ± 0.38[f,g]	1.02 ± 0.01[f-k]	3.72 ± 0.27[f-h]	4.73 ± 0.26[i-m]	0.27 ± 0.02[c-h]
PTN1	White	16.67 ± 0.10[l,m]	77.68 ± 1.32[a-d]	0.92 ± 0.04[f-i]	3.28 ± 0.06[e-g]	4.20 ± 0.10[g-k]	0.28 ± 0.01[d-h]
M7881	White	16.26 ± 0.08[m]	78.97 ± 0.59[d-e]	0.95 ± 0.02[f-j]	2.18 ± 0.02[a]	3.13 ± 0.04l[a-d]	0.44 ± 0.01[i-k]
MU2-42	Purple	15.03 ± 0.11[n]	79.45 ± 0.05[d-f]	1.44 ± 0.03[m,n]	3.81 ± 0.16[g,h]	5.25 ± 0.13[m]	0.38 ± 0.02[g-j]
SL	White	14.86 ± 0.02[n,o]	83.80 ± 1.06[h-j]	1.05 ± 0.06[g-k]	2.18 ± 0.18[a]	3.23 ± 0.24[a-e]	0.48 ± 0.01[j,k]
909-10-3	Purple	14.67 ± 0.06[n-p]	77.57 ± 1.20[a-d]	1.15 ± 0.05[i-m]	3.62 ± 0.17[f,h]	4.77 ± 0.22[j-m]	0.32 ± 0.00[f-i]
JHN	Purple	14.66 ± 0.03[n-p]	83.40 ± 1.48[h-j]	0.90 ± 0.01[f-i]	2.47 ± 0.01[a-c]	3.36 ± 0.02[a-f]	0.36 ± 0.00[f-j]
HCS	White	14.42 ± 0.03[o-q]	84.11 ± 0.21[i,j]	0.41 ± 0.02[a,b]	2.72 ± 0.04[a-e]	3.13 ± 0.02[a-d]	0.15 ± 0.01[a-c]
HLN	Purple	14.26 ± 0.02[p,q]	78.12 ± 0.01[b-e]	1.24 ± 0.01[j-m]	3.75 ± 0.25[f-h]	4.99 ± 0.25[l,m]	0.33 ± 0.02[f-i]
RB	Purple	13.96 ± 0.02[q]	79.44 ± 0.22[d-f]	1.37 ± 0.34[l-n]	2.53 ± 0.10[a-d]	3.90 ± 0.24[e-h]	0.55 ± 0.16[k]
SYN	Purple	13.16 ± 0.14[r]	79.13 ± 1.06[d-f]	1.01 ± 0.01[f-j]	3.82 ± 0.04[g,h]	4.82 ± 0.04[k-m]	0.26 ± 0.00[c-g]
NHN	White	7.83 ± 0.01[s]	84.12 ± 0.23[i,j]	1.10 ± 0.34[h-l]	3.68 ± 0.15[f-h]	4.77 ± 0.19[j-m]	0.30 ± 0.10[f-h]
KH	Purple	6.70 ± 0.01[t]	76.32 ± 0.47[a,b]	1.32 ± 0.02[k-n]	4.12 ± 0.27[h]	5.43 ± 0.28[m]	0.32 ± 0.02[f-i]
MU1-2313	Purple	6.75 ± 0.08[t]	76.96 ± 0.16[a-c]	1.55 ± 0.01[n]	5.28 ± 0.16[i]	6.83 ± 0.16[n]	0.29 ± 0.01[e-h]
RD6	White	6.23 ± 0.01[t]	84.18 ± 0.26[i,j]	0.62 ± 0.01[e-h]	2.37 ± 0.04[a-c]	2.99 ± 0.03[a-e]	0.35 ± 0.08[f-i]

Results are the means ± SE on dry basis and expressed as the percentage of whole grain rice flour. Values with different letters in the same column are significantly different with $P < 0.05$. [a]Dietary fibre: soluble dietary fibre (SDF), insoluble dietary fibre (IDF), calculated total dietary fibre (TDF = sum of SDF and IDF).

Table S4. Chemical compositions of milled bran samples.

Varieties	Colour	Fat	Starch	^{a}Dietary fibre			
				SDF	IDF	TDF	SDF:IDF
66B09	Brown	21.05 ± 0.04^{j,k}	27.74 ± 1.17^{p}	1.30 ± 0.02^{a-c}	31.93 ± 1.26^{h-j}	33.23 ± 1.28^{f-j}	0.04 ± 0.00^{a-c}
PK4#20A09	Brown	25.02 ± 0.10^{n}	6.78 ± 0.04^{a}	2.04 ± 0.17^{c-g}	33.80 ± 0.16^{k,l}	35.85 ± 0.00^{l-n}	0.06 ± 0.01^{b-f}
16F35	Brown	18.41 ± 0.05^{c,d}	26.74 ± 1.33^{o,p}	1.71 ± 0.38^{a-e}	32.20 ± 0.14^{i,j}	33.91 ± 0.52^{h-k}	0.05 ± 0.01^{a-d}
PK4#117A08	Brown	20.32 ± 0.24^{h-j}	21.61 ± 0.58^{i-k}	1.74 ± 0.27^{b-e}	31.44 ± 0.26^{g-j}	33.18 ± 0.52^{e-j}	0.06 ± 0.01^{a-e}
PK4#78A03	Brown	19.53 ± 0.02^{f-h}	18.53 ± 1.79^{d-g}	3.84 ± 0.19^{k-m}	27.22 ± 0.54^{b,c}	31.06 ± 0.72^{b-d}	0.14 ± 0.00^{k-l}
DGR	Brown	20.91 ± 0.57^{j,k}	15.49 ± 0.47^{c}	0.92 ± 0.01^{a,b}	31.26 ± 0.02^{g-j}	32.17 ± 0.03^{d-h}	0.03 ± 0.00^{a}
M9997	Brown	19.61 ± 1.24^{f-i}	21.47 ± 0.46^{i-k}	2.47 ± 0.26^{e-i}	29.04 ± 0.39^{d-f}	31.50 ± 0.50^{b-f}	0.09 ± 0.01^{e-h}
BMT	Brown	18.99 ± 0.76^{e,f}	23.79 ± 0.57^{k-n}	0.78 ± 0.07^{a}	26.83 ± 0.54^{b}	27.61 ± 0.47^{a}	0.03 ± 0.00^{a}
KTH	Brown	22.80 ± 0.01^{m}	18.72 ± 0.18^{e-h}	2.69 ± 0.24^{f-j}	28.75 ± 0.32^{c-e}	31.44 ± 0.56^{b-f}	0.09 ± 0.01^{f-i}
RD43	Brown	16.42 ± 0.98^{b}	19.38 ± 0.32^{g-i}	3.16 ± 0.02^{h-k}	34.17 ± 0.72^{l}	37.33 ± 0.70^{n-p}	0.09 ± 0.00^{f-i}
RD15	Brown	19.78 ± 0.08^{f-i}	16.34 ± 1.33^{c,d}	4.45 ± 0.29^{l-m}	25.25 ± 0.18^{a}	29.70 ± 0.47^{b}	0.18 ± 0.01^{m}
PNL80	Brown	21.97 ± 0.47^{l-m}	24.39 ± 0.30^{m,n}	1.51 ± 0.03^{a-d}	30.27 ± 0.58^{e-g}	31.79 ± 0.55^{c-g}	0.05 ± 0.00^{a-d}
PK1	Brown	21.20 ± 0.17^{j-l}	12.80 ± 0.04^{b}	3.11 ± 0.12^{h-k}	29.17 ± 0.05^{d-f}	32.28 ± 0.08^{d-i}	0.11 ± 0.00^{h-j}
KDML105	Brown	20.83 ± 0.02^{j,k}	24.01 ± 0.44^{l-n}	3.59 ± 0.38^{j-l}	34.36 ± 0.07^{l}	37.94 ± 0.31^{o,p}	0.10 ± 0.01^{h-j}
HS	Brown	17.64 ± 0.01^{c}	23.97 ± 0.69^{l-n}	1.59 ± 0.08^{a-e}	34.16 ± 1.00^{l}	35.75 ± 0.92^{k,n}	0.05 ± 0.00^{a-c}
PTN1	Brown	20.98 ± 0.49^{j,k}	22.17 ± 0.85^{j-m}	2.47 ± 0.08^{e-i}	30.74 ± 0.55^{g-i}	33.22 ± 0.63^{f-j}	0.08 ± 0.00^{d-h}
M7881	Brown	22.48 ± 0.01^{m}	22.06 ± 0.55^{j-l}	3.59 ± 0.02^{j-l}	27.75 ± 0.32^{b-d}	31.34 ± 0.34^{b-e}	0.13 ± 0.00^{j-l}
MU2-42	Purple	22.50 ± 0.46^{m}	16.09 ± 0.80^{c}	2.31 ± 0.10^{d-h}	29.10 ± 0.33^{d-f}	31.42 ± 0.43^{b-f}	0.08 ± 0.00^{d-h}
SL	Brown	22.01 ± 0.22^{l,m}	16.25 ± 0.31^{c,d}	2.71 ± 0.21^{g-j}	30.85 ± 0.15^{g-j}	33.56 ± 0.36^{g-j}	0.09 ± 0.01^{e-h}
909-10-3	Purple	21.26 ± 0.11^{k,l}	16.90 ± 1.73^{c-f}	3.28 ± 0.15^{i-k}	32.22 ± 1.32^{i-j}	35.50 ± 1.47^{k-n}	0.10 ± 0.00^{g-j}
JHN	Purple	21.14 ± 0.03^{j-l}	21.02 ± 0.56^{h-j}	4.47 ± 0.33^{l-m}	29.08 ± 0.15^{d-f}	33.55 ± 0.19^{g-j}	0.15 ± 0.01^{l,m}
HCS	Brown	18.91 ± 0.13^{e,f}	18.88 ± 0.02^{f-h}	4.56 ± 0.38^{m}	30.47 ± 0.24^{f-h}	35.03 ± 0.63^{j-m}	0.15 ± 0.01^{k-m}
HLN	Purple	20.48 ± 0.01^{i-k}	25.00 ± 0.11^{n,o}	2.00 ± 0.04^{c-g}	28.08 ± 0.24^{b-d}	30.08 ± 0.19^{b,c}	0.07 ± 0.00^{c-g}
RB	Purple	19.24 ± 0.02^{f,g}	19.65 ± 0.72^{g-i}	3.88 ± 0.92^{k-m}	32.34 ± 0.87^{j,k}	36.23 ± 0.05^{m-n}	0.12 ± 0.03^{j-k}
SYN	Purple	22.43 ± 0.37^{m}	16.53 ± 0.21^{c-e}	1.38 ± 0.07^{a-d}	39.71 ± 0.06^{m}	41.09 ± 0.13^{q}	0.03 ± 0.00^{a,b}
NHN	Brown	20.89 ± 0.01^{j,k}	16.99 ± 0.51^{c-f}	1.76 ± 0.24^{b-f}	29.94 ± 0.79^{e-g}	31.70 ± 1.03^{c-f}	0.06 ± 0.01^{a-e}
KH	Purple	12.98 ± 0.03^{a}	35.10 ± 0.16^{q}	3.73 ± 0.42^{k-m}	34.88 ± 0.08^{l}	38.61 ± 0.34^{p}	0.11 ± 0.01^{h-j}
MU1-2313	Purple	15.75 ± 0.01^{b}	22.45 ± 0.78^{j-m}	3.17 ± 0.18^{h-k}	39.30 ± 0.25^{m}	42.47 ± 0.43^{q}	0.08 ± 0.00^{d-h}
RD6	Brown	19.90 ± 0.17^{g-i}	17.81 ± 1.59^{c-g}	3.79 ± 0.97^{k-m}	30.32 ± 0.25^{f,g}	34.11 ± 1.22^{i-l}	0.13 ± 0.03^{i-l}

Results are the means ± SE on dry basis and expressed as the percentage of rice bran powder. Values with different letters in the same column are significantly different with $P < 0.05$. ^{a}Dietary fibre: soluble dietary fibre (SDF), insoluble dietary fibre (IDF), calculated total dietary fibre (TDF = sum of SDF and IDF).

Table S5. Pearson's correlation matrix: correlation coefficients of amylose content, gelatinization temperature, textural properties, and dietary fibre profiles.

	AC	GT	HRD	ADH	SPR	COH	GUM	CHEW	SDFWGR	IDFWGR	SDF:IDF
**AC**	1										
**GT**	-0.23	1									
**HRD**	**0.80****	-0.05	1								
**ADH**	**-0.75****	0.21	**-0.66****	1							
**SPR**	**0.76****	-0.25	**0.54****	**-0.61****	1						
**COH**	**0.78****	-0.25	**0.66****	**-0.61****	**0.86****	1					
**GUM**	**0.86****	-0.12	**0.96****	**-0.69****	**0.71****	**0.84****	1				
**CHEW**	**0.89****	-0.15	**0.87****	**-0.69****	**0.86****	**0.91****	**0.96****	1			
**SDFWGR**	**-0.63****	-0.02	**-0.70****	**0.45***	-0.35	**-0.58****	**-0.72****	**-0.62****	1		
**IDFWGR**	-0.25	0.25	0.05	0.29	-0.28	-0.22	-0.07	-0.15	0.25	1	
**SDF:IDF**	**-0.51****	-0.16	**-0.74****	0.31	-0.22	**-0.45***	**-0.69****	**-0.55****	**0.87****	-0.24	1

* Correlation is significant at the 0.05 level; ** Correlation is significant at the 0.01 level.

AC = amylose content; GT = gelatinization temperature; HRD = hardness; ADH = adhesiveness; SPR = springiness; COH = cohesiveness; GUM = gumminess; CHEW = chewiness; SDFWGR = SDF in whole grain rice; IDFWGR = IDF in whole grain rice; SDF:IDF = SDF to IDF ratio of whole grain rice.

Table S6. Texture profile analysis of cooked whole grain rice samples.

**Varieties**	**Hardness (HRD) (N)**	**Adhesiveness (ADH) (mN.sec)**	**Springiness (SPR) (Sec/Sec)**	**Cohesiveness (COH) (N.sec/N.sec)**	**Gumminess (GUM) (N)**	**Chewiness (CHEW) (N)**
66B09	30.50 ± 1.03^{c,d}	12.62 ± 1.58^{a,b}	0.65 ± 0.04^{c-g}	0.47 ± 0.00^{b,c}	13.27 ± 0.84^{b,d}	8.67 ± 0.96^{d,e}
PK4#20A09	36.67 ± 0.17^{a}	10.62 ± 1.42^{a}	0.66 ± 0.01^{c-f}	0.44 ± 0.04^{c-f}	16.00 ± 1.75^{a}	10.61 ± 1.31^{a-c}
16F35	30.41 ± 0.95^{c,d}	14.34 ± 0.27^{a-c}	0.70 ± 0.01^{b,c}	0.49 ± 0.01^{b}	14.93 ± 0.13^{a,b}	10.62 ± 0.44^{a,b}
MU2-00005	27.62 ± 0.16^{e,f}	12.93 ± 1.46^{a,b}	0.70 ± 0.04^{b,c}	0.50 ± 0.01^{a,b}	13.91 ± 0.33^{b,c}	9.96 ± 0.70^{a-d}
PK4#117A08	34.44 ± 0.16^{b}	13.41 ± 2.25^{a,b}	0.67 ± 0.01^{b-d}	0.46 ± 0.01^{b-d}	15.78 ± 0.21^{a}	10.66 ± 0.07^{a,b}
PK4#78A03	29.04 ± 0.68^{d,e}	11.81 ± 2.79^{a}	0.70 ± 0.01^{b,c}	0.39 ± 0.01^{f-l}	11.42 ± 0.69^{e-g}	8.01 ± 0.64^{e,f}
DGR	25.00 ± 0.42^{g-i}	19.30 ± 4.09^{a-e}	0.74 ± 0.01^{b}	0.47 ± 0.01^{b-d}	12.70 ± 0.54^{d-f}	9.61 ± 0.26^{b,c}
M9997	24.02 ± 0.18^{h-j}	24.00 ± 7.38^{a-e}	0.89 ± 0.02^{a}	0.53 ± 0.00^{a}	12.84 ± 0.25^{c-e}	11.52 ± 0.08^{a}
BMT	24.59 ± 1.01^{g-i}	12.22 ± 1.50^{a}	0.67 ± 0.04^{b-e}	0.41 ± 0.01^{e-i}	10.07 ± 0.28^{g,h}	6.74 ± 0.55^{f,g}
KTH	31.59 ± 0.39^{c}	47.64 ± 2.96^{f-j}	0.67 ± 0.03^{b-d}	0.46 ± 0.00^{b-d}	14.63 ± 0.22^{a,b}	9.86 ± 0.56^{b-d}
RD43	25.77 ± 0.38^{f-h}	19.65 ± 0.45^{a-e}	0.58 ± 0.04^{g-i}	0.44 ± 0.02^{c-e}	11.39 ± 0.35^{e-g}	6.58 ± 0.67^{f,g}
RD15	26.23 ± 0.08^{fg}	21.70 ± 3.70^{a-e}	0.58 ± 0.02^{g-i}	0.42 ± 0.01^{d-g}	11.08 ± 0.36^{f,g}	6.43 ± 0.48^{f,g}
PNL80	22.27 ± 0.32^{jk}	56.58 ± 0.11^{h-k}	0.64 ± 0.03^{c-g}	0.41 ± 0.02^{e-i}	9.20 ± 0.46^{h,i}	6.00 ± 0.50^{g,h}
PK1	18.70 ± 0.05^{m,n}	17.91 ± 1.62^{a-d}	0.64 ± 0.06^{c-g}	0.38 ± 0.01^{g-k}	7.19 ± 0.23^{j,k}	4.62 ± 0.62^{h-k}
KDML105	20.63 ± 0.16^{k,l}	28.10 ± 4.79^{a-f}	0.58 ± 0.01^{e-h}	0.39 ± 0.01^{g-j}	8.12 ± 0.21^{i-k}	4.75 ± 0.04^{h-j}
HS	19.39 ± 0.87^{l-n}	47.13 ± 1.39^{f-j}	0.53 ± 0.01^{h-k}	0.36 ± 0.01^{j-l}	7.06 ± 0.56^{j,k}	3.78 ± 0.37^{i-l}
HD	23.11 ± 1.25^{i,j}	77.14 ± 11.22^{k}	0.58 ± 0.03^{g-i}	0.39 ± 0.01^{g-j}	9.19 ± 0.74^{h,i}	5.28 ± 0.70^{g-i}
PTN1	18.39 ± 0.04^{n}	62.22 ± 9.35^{j,k}	0.55 ± 0.02^{h-j}	0.37 ± 0.01^{i-k}	6.84 ±0.10^{k-m}	3.83 ± 0.21^{i-l}
M7881	14.19 ± 0.60^{o,p}	35.57 ± 7.97^{c-h}	0.58 ± 0.01^{f-i}	0.38 ± 0.00^{h-k}	5.35 ± 0.17^{l-n}	3.14 ± 0.14^{k-m}
MU2-42	16.05 ± 0.04^{o}	55.51 ± 12.00^{g-k}	0.48 ± 0.01^{j,k}	0.32 ± 0.01^{l,m}	5.21 ± 0.11^{m,n}	2.48 ± 0.02^{l,m}
SL	22.14 ± 0.62^{j,k}	39.38 ± 6.78^{d-i}	0.54 ± 0.04^{h-k}	0.39 ± 0.02^{g-j}	8.66 ± 0.20^{h-j}	4.66 ± 0.45^{h-k}
909-10-3	20.98 ± 1.19^{k,l}	34.15 ± 7.68^{b-g}	0.49 ± 0.03^{j,k}	0.31 ± 0.02^{m}	6.64 ± 0.73^{k-m}	3.26 ± 0.57^{j-m}
JHN	20.58 ± 0.45^{k,m}	60.24 ± 7.99^{j,k}	0.55 ± 0.02^{h-j}	0.36 ± 0.01^{j-l}	7.49 ± 0.35^{j,k}	4.14 ± 0.31^{i-k}
HCS	19.09 ± 1.00^{l-n}	54.37 ± 25.53^{g-j}	0.50 ± 0.02^{i-k}	0.36 ± 0.02^{j-l}	7.00 ± 0.68^{j-l}	3.52 ± 0.50^{j-m}
HLN	20.77 ± 1.07^{k,l}	37.08 ± 7.22^{d-h}	0.53 ± 0.03^{h-k}	0.34 ± 0.01^{k-m}	7.19 ± 0.60^{j,k}	3.85 ± 0.52^{i-l}
RB	13.04 ± 0.18^{p}	46.24 ± 7.98^{f-j}	0.46 ± 0.03^{k}	0.34 ± 0.02^{k-m}	4.47 ± 0.38^{n}	2.09 ± 0.30^{m}
SYN	23.53 ± 0.94^{i,j}	40.22 ± 1.23^{e-i}	0.60 ± 0.00^{d-h}	0.41 ± 0.01^{e-h}	9.77 ± 0.73^{g-i}	5.95 ± 0.40^{g,h}

Results are the means ± SE. Values with different letters in the same column are significantly different with $P < 0.05$. The texture analyzer instrumental parameters including hardness (HRD), adhesiveness (ADH), springiness (SPR), cohesiveness (COH), gumminess (GUM), and chewiness (CHEW) were derived from Texture analyzer (TA-XT plus, Stable Micro System, Godalming, UK) with two-cycle compression test.

Table S7. Genotype of rice samples: the GT/TT polymorphism at the 5′ splice site of the first intron of the waxy *(Wx)* gene and two predominant *Wx* alleles.

**Varieties**	***Wx* Splice site**	***Wx* alleles**
66B09	GT	Wx^a
PK4#20A09	GT	Wx^a
16F35	GT	Wx^a
MU2-00005	GT	Wx^a
PK4#117A08	GT	Wx^a
PK4#78A03	GT	Wx^a
DGR	GT	Wx^a
M9997	GT	Wx^a
BMT	GT	Wx^a
KTH	GT	Wx^a
RD43	TT	Wx^b
RD15	TT	Wx^b
PNL80	TT	Wx^b
PK1	TT	Wx^b
KDML105	TT	Wx^b
HS	TT	Wx^b
HD	TT	Wx^b
PTN1	TT	Wx^b
M7881	TT	Wx^b
MU2-42	TT	Wx^b
SL	TT	Wx^b
909-10-3	TT	Wx^b
JHN	TT	Wx^b
HCS	TT	Wx^b
HLN	TT	Wx^b
RB	TT	Wx^b
SYN	TT	Wx^b

Table S8. Range of basic nutrient compositions of whole grain rice, milled rice, and rice bran.

Nutrient	Whole grain rice	Milled rice	Rice bran
Starch	75.8-84.6	86.9-92.9	6.8-35.1
Lipids	2.9-3.4	0.4-0.9	13.0-25.0
Protein	7.3-8.8	7.6-9.0	16.8-20.1
Dietary fibre	2.9-6.8	1.2-2.6	27.6.-42.5
Ash	1.4-1.6	0.4-0.5	9.7-11.4

Results are the percentage of whole grain rice flour or milled rice flour or rice bran powder

Table S9. Comparison of the β-glucan and pectin content in whole grain rice and rice bran.

Varieties	β-glucan		Pectin	
	^{a}**Grain**	^{b}**Bran**	^{a}**Grain**	^{b}**Bran**
PK4#20A09	0.03 ± 0.00^{a}	$0.24 \pm 0.01^{a\text{-}d}$	0.07 ± 0.01^{a}	0.26 ± 0.01^{a}
PK4#78A03	0.03 ± 0.00^{a}	$0.25 \pm 0.00^{b\text{-}d}$	$0.10 \pm 0.01^{a,b}$	0.27 ± 0.01^{a}
RD43	0.03 ± 0.00^{a}	$0.24 \pm 0.01^{a\text{-}d}$	$0.16 \pm 0.03^{b\text{-}d}$	0.31 ± 0.01^{a}
RD15	$0.06 \pm 0.02^{a\text{-}c}$	0.29 ± 0.00^{e}	0.25 ± 0.00^{e}	0.29 ± 0.02^{a}
PK1	$0.07 \pm 0.01^{b\text{-}c}$	$0.22 \pm 0.00^{a\text{-}c}$	$0.11 \pm 0.00^{a,b}$	0.30 ± 0.04^{a}
M7881	$0.08 \pm 0.00^{b,c}$	0.21 ± 0.00^{a}	$0.14 \pm 0.04^{b,c}$	0.29 ± 0.06^{a}
SLK	0.11 ± 0.00^{d}	$0.26 \pm 0.00^{d,e}$	$0.16 \pm 0.01^{b\text{-}d}$	$0.33 \pm 0.04^{a,b}$
JHN	0.11 ± 0.00^{d}	$0.26 \pm 0.02^{c\text{-}e}$	0.27 ± 0.01^{e}	$0.32 \pm 0.01^{a,b}$
HCS	$0.09 \pm 0.01^{c,d}$	$0.23 \pm 0.01^{a\text{-}c}$	$0.16 \pm 0.01^{b\text{-}d}$	0.27 ± 0.01^{a}
RB	0.14 ± 0.02^{e}	0.40 ± 0.03^{f}	0.25 ± 0.04^{e}	0.42 ± 0.02^{b}

Results are the means ± SE on dry basis. Values with different letters in the same column are significantly different with $P < 0.05$. β-glucan and pectin content are presented as the percentage of ^{a}whole grain rice flour or ^{b}milled bran powder.
